# Baiting studies on oral vaccination of the greater kudu (*Tragelaphus strepsiceros*) against rabies

**DOI:** 10.1007/s10344-018-1220-z

**Published:** 2018-10-09

**Authors:** Rainer Hassel, Steffen Ortmann, Peter Clausen, Mark Jago, Floris Bruwer, Pauline Lindeque, Conrad Freuling, Ad Vos, Thomas Müller

**Affiliations:** 1 School of Veterinary Medicine, University of Namibia, Private Bag 13301, Windhoek, Namibia; 2 IDT Biologika GmbH, Am Pharmapark, 06861 Dessau-Rosslau, Germany; 3 Okosongoro Safari Ranch, P.O. Box 324, Omaruru, Namibia; 4 Agra ProVision, Agra Limited, Private Bag 12011, Windhoek, Namibia; 5 Institute of Molecular Virology and Cell Biology, Friedrich-Loeffler-Institute, 17493 Greifswald - Insel Riems, Germany

**Keywords:** Bait development, Bait distribution, Greater kudu, Oral vaccination, Rabies

## Abstract

Rabies in the greater kudu (*Tragelaphus strepsiceros*), one of the largest African antelopes, is a phenomenon unique to Namibia. Since the mid-1970s, the country has been plagued by two epizootics that claimed thousands of casualties among the indigenous kudu population. Reasons as to why kudus appear to be exceptionally susceptible to the disease still remain speculative at best. Because the Namibian economy relies heavily on trophy hunting, game meat hunting, and ecotourism, the current severe spread of rabies among kudus calls for effective measures to protect these antelopes. Oral vaccination of kudus may offer a possibility provided efficacious oral rabies vaccines are available. In this screening study, we explored options for attractive baits that facilitate optimal vaccine uptake by the target species and a bait distribution system that maximizes bait availability to the target species meanwhile minimizing bait depletion by non-target species. The results show that gelatin-based baits mixed with local (pods of the camel thorn tree) but also imported (apple-flavored corn meal) attractants are highly attractive for kudus providing a basis for future oral rabies vaccine baits.

**Electronic supplementary material:**

The online version of this article (10.1007/s10344-018-1220-z) contains supplementary material, which is available to authorized users.

## Introduction

Namibia is a country rich in a wide range of wildlife resources attracting thousands of tourists annually from all over the world. The Greater kudu, *Tragelaphus strepsiceros*, a large African herbivore, occupies the browser-trophic niche in southern Africa (Conybeare 1975; Owen-Smith 1979; Owen-Smith and Cooper 1985, 1989; Wilson 1970) and is the one most popular and iconic species of antelope in Namibia. However, since 1977, two epizootics of rabies occurred in the Greater kudu. While between 1977 and 2017, a total of 1049 cases of rabies were laboratory confirmed in this antelope species, the number of unrecorded cases is many times higher. During the first epizootic from 1977 to 1986, approximately 30,000 to 50,000 kudus or 20–40% of the total population are believed to have died of rabies (Barnard and Hassel 1981; Hassel 1982; Hübschle 1988; Schneider 1985), and there is no indication that the ongoing intermittent outbreaks of the disease will subside any time soon.

Livestock farming is one of the most important sources of income and is highly regarded in the Namibian society. In addition to cattle and sheep farming, farming of kudus, the second-largest African antelope species, is an important economic branch. As one of the major game species for meat production and a highly priced trophy species, this antelope is very popular as a farmed game species across the country. Namibia hopes to promote its game meat production industry and exports to regional and international markets by 2020, after game was identified as a niche market with potential to grow. It is therefore understandable that in contrast to other African countries, a rabies outbreak among the kudu population can have a significant negative economic and health impact on the farming community of Namibia. Therefore, it has become critical to investigate pathways for effective rabies control in kudus in Namibia (Scott et al. 2012).

While rabies vaccination of kudus by intramuscular injection is an entirely impractical option in terms of extremely high costs, work load, and animal welfare issues (stress induced injuries and/or mortality), oral rabies vaccination as developed successfully for wild carnivores ((Rupprecht et al. 2008) could offer a unique opportunity to cost-effectively prevent rabies in this species. This assumption is supported by experimental studies in wild ruminants like white-tailed deer (*Odocoileus virginianus*) and red deer (*Cervus elaphus*) showing that oral administration with BCG vaccine and heat-inactivated *Mycobacterium bovis*, respectively, is able to induce an immune response against bovine tuberculosis (Lopez et al. 2016; Nol et al. 2008; Palmer et al. 2014b). Experimental studies resulted in commercial vaccines for oral administration in ruminants for immunization of calves in controlling diarrhea caused by bovine rotavirus and coronavirus. While these vaccines are taken up in the gastrointestinal tract (de Leeuw and Tiessink 1985), in contrast, for oral vaccination against rabies using attenuated virus constructs, one needs to target the oral cavity (Baer et al. 1971, 1975).

In principle, oral rabies vaccines developed for wild carnivores could also be used in a herbivorous species. Important preconditions, however, are that they possess a high safety profile and that a minimum effective dose has been established in experimental studies. Recently, it was shown that in principle, kudus can be vaccinated by the oral route and protected against a subsequent rabies infection, although further studies need to be initiated to optimize oral vaccine uptake and delivery (Hassel et al., unpublished). Next to efficacious oral rabies vaccines, another considerable challenge is the development of suitable baits. Baits for delivery of vaccines and other pharmaceuticals have been developed for wild carnivores (Johnston and Voigt 1982; Linhart et al. 2002; MacInnes 1988; Schneider et al. 1987; Vos et al. 2004), dogs (Matter et al. 1995; Schuster et al. 1998), feral swine (Ballesteros et al. 2011; Campbell and Long 2007; Fletcher et al. 1990; Kaden et al. 2000; Kavanaugh and Linhart 2000; Snow et al. 2016), and herbivorous species such as white-tailed deer (Palmer et al. 2014a, b) and prairie dogs (Abbott et al. 2018; Tripp et al. 2014) but not yet for kudus. Therefore, the objectives of this study was trying (i) to explore potential suitable baits for kudus that are attractive to and well accepted by the target species and facilitate optimal vaccine uptake and (ii) to identify a possible bait distribution system that maximizes bait availability to the target species meanwhile minimizing bait depletion by non-target species.

## Material and methods

### Animal and housing conditions

The bait studies were an add-on study in the frame of experimental transmission and vaccination studies in kudu (Hassel et al., unpublished). A total of 19 adult free-living kudus were caught by mass capture and by individual darting from a helicopter on three conservancies in Northern Namibia and transported to the experimental holding facility (“boma”) at Okosongoro Safari Ranch in the Omaruru District. The site was enclosed by a 3.2-m high fence and guarded 24 h per day. Kudus were housed in pens of 7 m × 14 m in size consisting of a covered and an open area, which could be separated from each other by a sliding door and entered separately. Upon arrival, kudus received parasite treatment with doramectin (Dectomax—1% solution, Pfizer, Sandton, South Africa) as well as multivitamins and metabolic stimulant injections (Kyrophos Metabolic-V, Kyron Laboratories, Johannesburg, South Africa). Animals were fed two times a day. The diet consisted of lucerne straw and standard game cubes, alternated with game cubes plus ivermectin (Feedmaster, Windhoek, Namibia; Boskos WESenterprises, Thabazimbi, South Africa). Depending on availability (hot dry season), the diet was supplemented with pods of the camel thorn tree (*Acacia erioloba*) and umbrella thorn tree (*Acacia tortilis*). The pens were cleaned daily; drinking water was offered ad libitum and supplemented with regular addition of probiotics, minerals, and vitamins. The baiting studies were conducted under general permit nos. 101631, 101835, 101826, and 101825 for capturing and keeping of game animals and research permit nos. 1984/2014 and 2152/2016 under the Nature Conservation Ordinance 4 of 1975 issued by the Ministry of Environment and Tourism. Transport of animals from the capture site to the experimental facility was secured by a Veterinary Movement Permit of the Directorate of Veterinary Services (DVS). The experimental facility conformed to the minimum requirements of the Ministry of Environment and Tourism for captive game and was approved by DVS.

### Baits

**Fig. 1:**
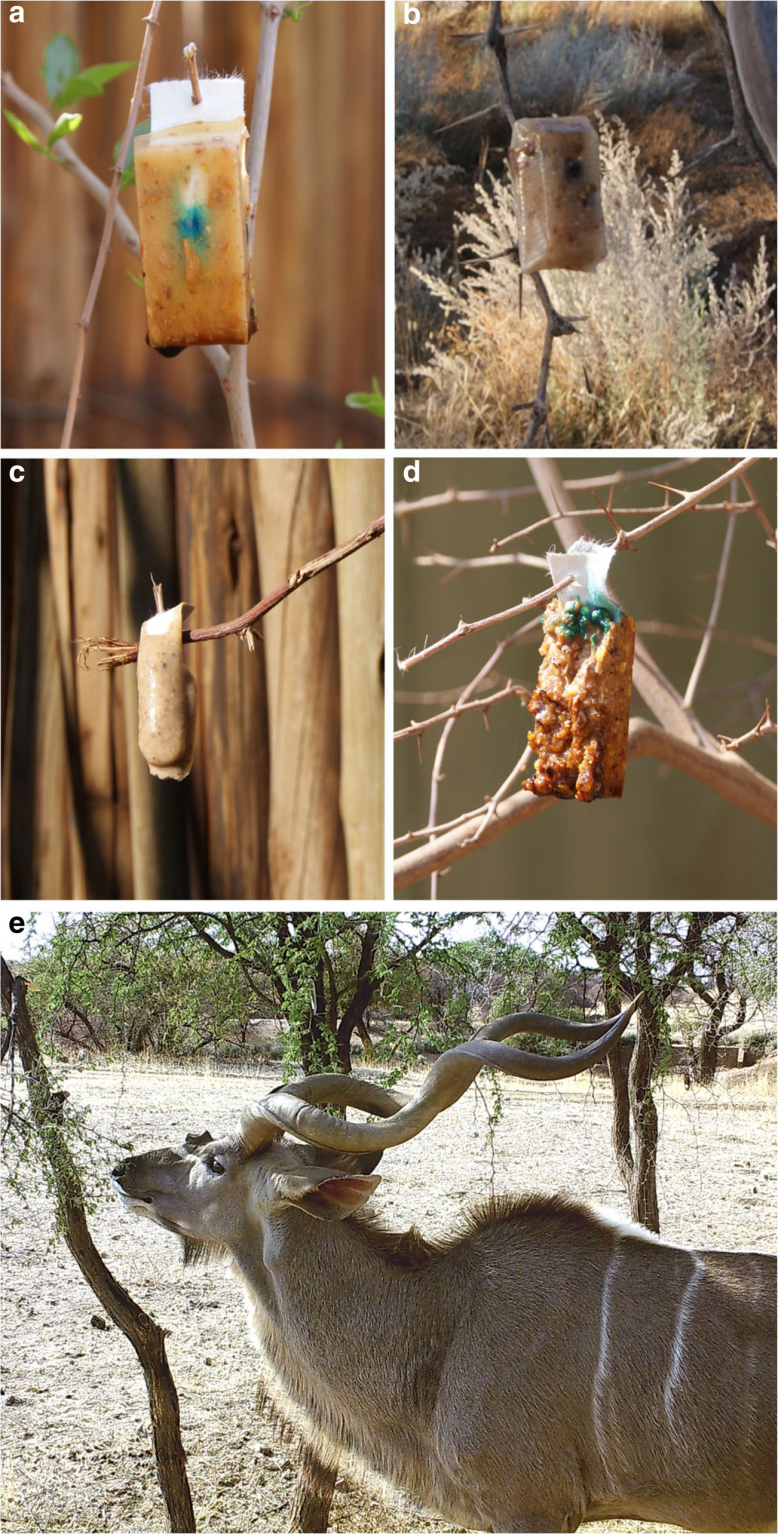
The gelatin-based experimental baits 2.5 × 4.5 × 1.0 cm in size. **a** Gelatin-based baits mixed with grinded pods of the camel thorn tree (type 1). **b** Gelatin-based baits containing pits of camel thorn tree pods (type 2). **c** Gelatin-based baits mixed with grinded pods of the camel thorn tree (type 1—dipped) and **d** apple-flavored corn meal baits (type 3). Bait uptake of a **e** free-roaming male kudu

The bait ingredients were carefully selected according to reported food preferences of kudus in the field. To this end, two experimental gelatin-based baits were prepared that were either mixed with grinded pods of the camel thorn tree (type 1) or contained pits of camel thorn tree pods (type 2), a favored browse plants of kudus (Owen-Smith and Cooper 1983). To explore the feasibility of using baits that would be easier to manufacture industrially, apple-flavored corn meal baits (type 3) were tested as well. The baits are approx. 2.5 cm × 4.5 cm × 1.0 cm in size (Fig. 1). Of the former grinded camel thorn tree pod bait (type 1), two different variants were used in a particular baiting study 4, one with a very thin layer of bait matrix (dipped) and one with a thicker layer of bait matrix (poured). As control baits, pods of the umbrella thorn tree (control bait 1) or pods of the camel thorn tree (control bait 2) were used (Online Resource 1).

### Baiting studies

Four different baiting studies were conducted to prove bait acceptability by kudus.

While baiting study 1 was drafted to assess the attractiveness of the different experimental baits (types 1–3), baiting studies 2–4 were designed to evaluate the bait distribution and potential vaccine delivery system.

To this end, in baiting study 1, in total, eight pieces of one of the three selected experimental baits were homogeneously mixed with eight control baits (umbrella thorn tree pods) (Online Resource 2) and placed in the food tray within two selected pens holding four female animals each (two-food-preference test). After several hours in accordance with the feeding routine, bait removal was recorded and a new set of baits was placed.

In baiting study 2 designed to assess bait uptake in captivity, in each pen, 24 baits were placed on the thorns of acacia bushes within the outside section of the two holding pens overnight including 8 baits each of types 2 and 3 and controls (camel thorn tree pods). The baits were divided over several bushes in such a way that every bush contained at least 2 baits of each type (Online Resource 3). The following morning bait disappearance was recorded. At this stage, the pens were not yet equipped with cameras, so the bait removal could only be assessed by indirect observations.

Baiting study 3 aimed at assessing bait uptake by free-roaming kudu. To this end, five baits of types 1 and 3 each as well as control baits (camel thorn tree pods) were placed on the thorns in each of the three acacia trees close to a waterhole (Online Resource 4) that was frequently visited by a large free-roaming male kudu antelope on the Okosongoro Safari Ranch (Fig. 1). The baits were pierced on the thorns in the early morning on three consecutive days, and bait uptake was monitored partially by camera trap (MINOX DTC 1000; Minox, Wetzlar, Germany) to allow undisturbed rapprochement and browsing by the male kudu. When the animal left the place, disappearance of baits was thoroughly checked at close range.

Finally, in study 4, uptake of vaccine sachet loaded baits was assessed. To this end, ten kudus used as naïve contact animals in a horizontal transmission experiment (Hassel et al., unpublished) were offered different baits containing a sachet made of biodegradable foil (size 4.6 × 1.6 × 0.7 cm) filled with distilled water. The animals were divided into four different groups (two groups of two animals and two groups of three animals each). The sachet-loaded baits were pierced on the thorns of acacia shrubs within the outdoor section of the enclosure. Two experimental baits were used: apple-flavored corn meal (type 3) and grinded camel thorn tree pods mixed with gelatin (type 1—dipped and poured). Finally, camel thorn tree pods (control bait 2) were scooped out and a vaccine sachet was placed inside (Online Resource 5).

Differences in bait preference in screening study 1 between the test and control baits were tested using Fisher’s *t* test (McDonald 2014). The statistical significance threshold *α* was set to 0.05. All captured animals were vaccinated against rabies and rehomed at the end of the study.

#### Data availability

All data generated or analyzed during this study are included in this published article [and its supplementary information files].

## Results

The two-food-preference test in bating study 1 showed that the acceptance of the experimental baits (Online Resources 1 and 2) for kudus kept in captivity was similar or even better compared to the pods of the umbrella thorn tree used as control baits (control bait 1). On one occasion only, the uptake of the gelatin-based baits containing grinded pods of the camel thorn tree (type 1) was significantly higher (Online Resources 1 and 2) (Table 1).

**Table 1:** Results of the two-food-preference test (baiting study 1) of three experimental bait types in comparison to umbrella thorn tree pods used as control baits. Experimental baits used were as follows: gelatin-based baits containing grinded pods of the camel thorn tree (type 1), gelatin-based baits containing pits of camel thorn tree pods (type 2), and apple-flavored corn meal baits (type 3)

		Pen 1 (*n*/*N*)		Pen 2 (*n*/*N*)	
Time period	Bait type	Test baits	Control bait	Test baits	Control bait
09:40–11:30	2	0/8	3/8	0/8	0/8
Fisher’s *t* test		*p* = 0.20		*p* = 1.00	
11:45–14:30	1	8/8	1/8	8/8	1/8
Fisher’s *t* test		*p* = 0.004*		*p* = 0.004*	
14:45–09:00	3	8/8	8/8		
	1			8/8	8/8
Fisher’s *t* test		*p* = 1.00		*p* = 1.00	

*n* baits removed, *N* baits placed

*Significant

In baiting study 2, during which the same animals had to locate the baits pierced on the thorns of the acacia bushes within the enclosure (Online Resource 3), all 24 baits in pen 1 were gone overnight and also no bait fragments were found on the floor. In pen 2, with the exception of three baits on the floor, one of each type, also here, all other baits had disappeared. During the first 2 days of bait study 3, when 5 apple-flavored corn meal baits (type 3) and gelatin-based baits with grinded camel thorn tree pods (type 2) each were distributed over 3 acacia trees (Online Resource 4), all baits were located and consumed by the free-roaming male kudu (Fig. 1). On a third occasion, none of the baits was taken and after several days, the dehydrated baits were removed.

The disappearance of the vaccine-loaded baits pierced on the thorns of the shrubs in baiting study 4 is summarized in Online Resource 6. Bait acceptance in all groups was very good, except for group C. All baits offered to the animals in this particular group were still present after 24 h. Hence, in a second attempt, the baits were replaced by five baits of each of the other three vaccine bait types offered to the other groups (Online Resource 6). Again, the animals in group C did not consume any of these baits within 24 h.

## Discussion

Within the Namibian agricultural and touristic sector, there is growing concern about the role kudus play in the maintenance and transmission of rabies. To successfully control the disease in this antelope, innovative disease management tools for kudus are needed. Expanding the concept of oral vaccination against rabies as successfully developed for wild carnivores to kudus seems to be a feasible approach as shown recently (Hassel et al., unpublished). This is the first time that baits for delivery of vaccines for kudus have been developed and tested experimentally. The bait ingredients were carefully selected according to reported food preferences of kudus in the field. Usually, kudus feed on different shrub and tree species but also small amounts of herbs and grass. Where shrubs, trees, and herbs are predominant, browse predominates the diet; however, when scarce, grass can be consumed in considerable amounts (Conybeare 1975; Hooimeijer et al. 2005). Acacia tree spp. including camel thorn and umbrella thorn, some of southern Africa’s most common trees, belong to the favored browse plants of kudus. Because the pods are highly nutritious and are actively sought when available in particular during the dry season (Owen-Smith and Cooper 1983), these pods were regarded the perfect attractant for our baits.

The experimental baits were well accepted by the kudus, also when distributed by the suggested delivery system: piercing the baits on thorns of acacia trees, the leaves of this tree are a natural food source for kudus. However, differences were clearly visible between individual animals. Bait uptake was lower in pen 2 than in pen 1 during the first screening study (Table 1); the amount of food intake of the former group of animals was generally low. Also, both animals in group C of study 4 did not take any of the baits containing the vaccine sachet, irrespective of the bait type. As these baits were almost all rapidly accepted by the animals in the other groups (A, B, D) (Online Resource 6), obviously, it was not the palatability of the baits but the animals in this group C that somehow did not eat the baits (incl. the control bait) (Online Resource 6). Unfortunately, acacia pods are not everywhere easily available and also grinding the pods is not an easy task. Therefore, with the apple-flavored bait, we were also looking for alternatives to baits made from local material. Interestingly, this particular bait was also well accepted by the animals. Since this bait type would be much easier to manufacture, the material needed can easily be purchased. Under field conditions (study 3), both baits made from local material as well as baits that can much more easily be produced en masse were well accepted, however, more intensified studies with the different baits should be carried out to identify the most optimal bait.

Next to a safe and efficacious oral rabies vaccine and an attractive bait, the third component of oral vaccination concerns a suitable bait distribution system. In Europe and North America, oral rabies vaccine baits are distributed predominantly by plane using a pre-determined bait density per square kilometer; distance between individual bait drops is fixed, as well as the distance between the flight lines (Müller et al. 2012). In certain areas like heavily populated areas, baits cannot be distributed by plane and are therefore distributed by hand. Sometimes, new strategies like clustered baiting using for example bait stations are used (Boulanger et al. 2006). These techniques are also applied for wild boars (*Sus scrofa*), a species with a similar social structure as kudus (Ballesteros et al. 2011; Rossi et al. 2015). Unfortunately, most of these bait distribution systems are not suitable for targeting kudus in Namibia. Hence, a system was tested that would reduce bait depletion by non-target species considerably, incl. insects and rodents. Also, the system to be developed should reduce exposure to the high temperatures and direct sun light that can be detrimental for the vaccine baits, both for the bait matrix (melting) and vaccine (titre). Piercing baits on thorns at a certain minimum height within the favored acacia trees by kudus (studies 2 and 3; Online Resources 3 and 4) could offer a suitable solution to bait depletion by non-target species, including smaller antelopes (browsers).

A similar distribution system by placing baits at a certain height in canopies of trees to avoid bait competition by ground-dwelling animals and over-flying birds has already been suggested for baiting gorillas against Ebola (Dolgin 2008). Furthermore, such a system reduces thermal stress for bait and vaccine while the canopy of the trees/shrubs in which the baits are placed protects it from direct sunlight. Also, placing the baits not on the ground between the grass vegetation but exposing them to air movement increases heat loss from the bait to the air by convection. Bait and vaccine uptake by larger browsers like eland (*Taurotragus oryx*) which share the range with kudus on many farms would not be disadvantageous, since this species has also been shown to be susceptible to rabies infection and would benefit from it. Due to the large areas affected by kudu rabies, optimal baiting strategies in terms of bait delivery and distribution (spatial and temporal) would need to be developed in the future. For example, to achieve optimal bait uptake, baiting could preferably be done during the dry season when the kudus actively visit the waterholes. It would also be necessary to have a number of baiting stations, preferably near waterholes, and to lure the animals by “pre-baiting” using camel thorn pods, to these stations. Also, based on the observation in baiting study 3, continued provisions of vaccine baits at baiting stations over several days need to be taken into account.

In conclusion, several candidate baits have been identified that were readily taken up by captive and free-living kudus. Finally, a bait distribution system has been suggested that maximizes uptake by the target population meanwhile minimizing uptake by non-target species. However, this bait distribution system must be developed in more detail under field conditions to assure high bait uptake and hence, high vaccination coverage among free-roaming kudus and also to qualitatively and quantitatively identify potential bait competitors.

## Electronic supplementary material

Online Resource 1 Camel thorn tree pods (left) and umbrella thorn tree pods (right) used as control baits (PDF 801 kb)

Online Resource 2 Preparation of 8 pieces of the gelatin-based baits mixed with grinded pods of the camel thorn tree (type 1, left) mixed with 8 control baits (umbrella thorn tree pods, right - control bait 1) to be homogenously mixed in the food tray in bating study 2 (PDF 848 kb)

Online Resource 3 Experimental baits pierced on the thorns of the acacia bushes for bait-uptake by free-roaming Kudu within the enclosure (PDF 865 kb)

Online Resource 4 Placebo baits were pierced on the thorns of an acacia tree (left) located at a waterhole close to the experimental facility. (PDF 1448 kb)

Online Resource 5 The vaccine sachet from biodegradable foil placed in the camel thorn tree pod (control bait 2) (PDF 133 kb)

Online Resource 6 (PDF 147 kb)
