# Supplementary figures and images for: Baiting studies on oral vaccination of the greater kudu (Tragelaphus strepsiceros) against rabies
Source: Eur J Wildl Res. 2018 Oct 9;64(6):62. doi: 10.1007/s10344-018-1220-z (PMC7088030; doi:10.1007/s10344-018-1220-z)

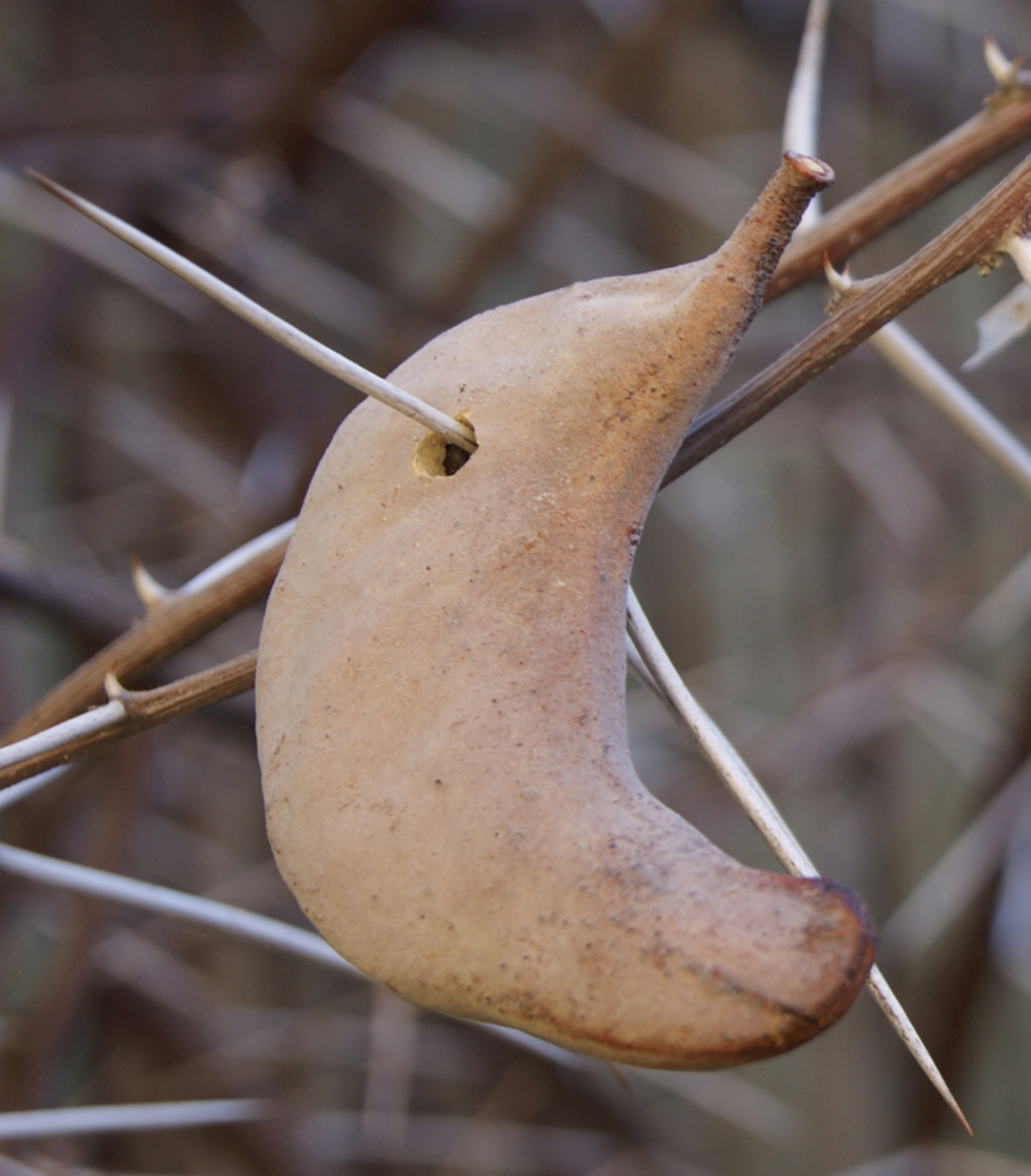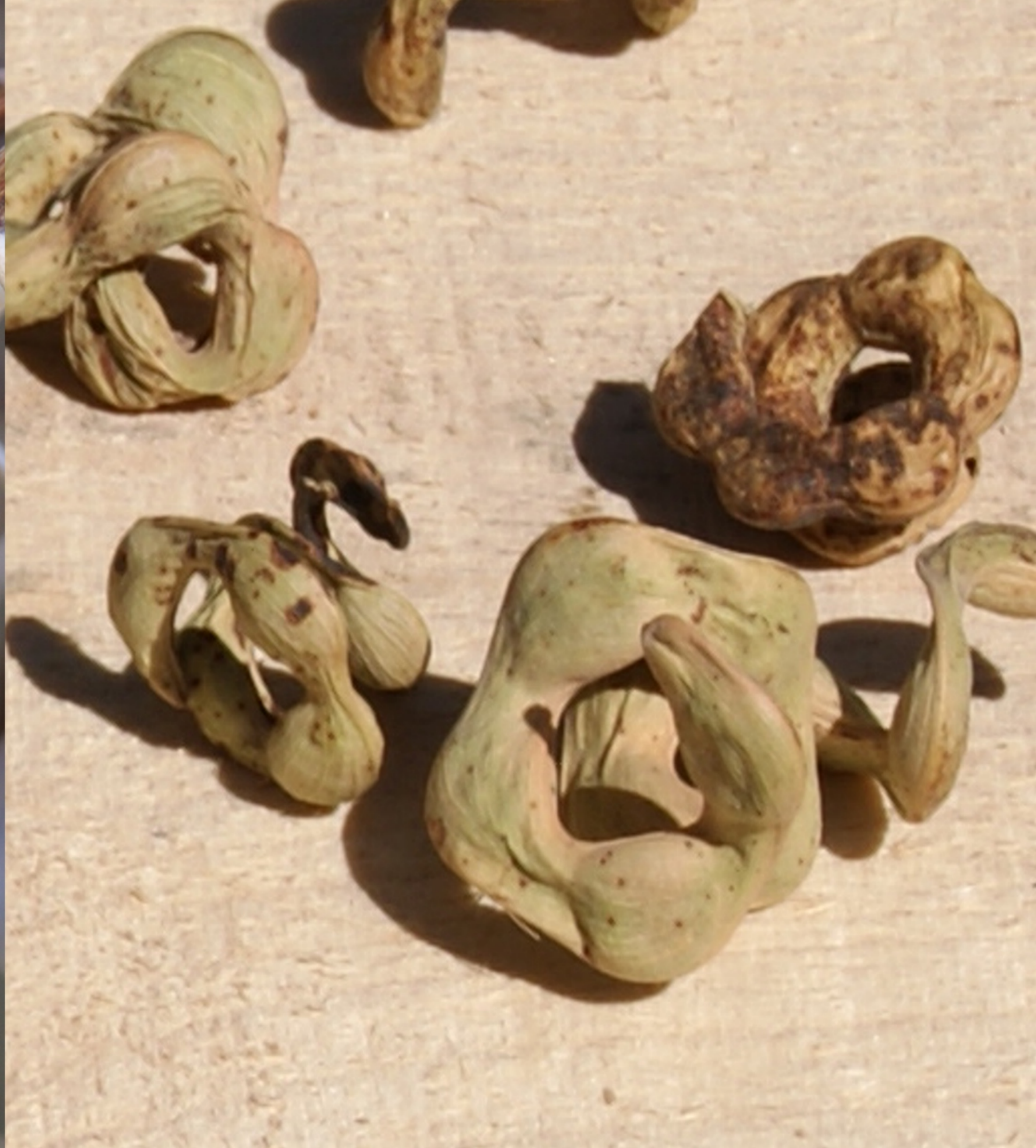

Supplement: Supplementary file 1 — Camel thorn tree pods (left) and umbrella thorn tree pods (right) used as control baits (PDF 801 kb) [file 10344_2018_1220_MOESM1_ESM.pdf]

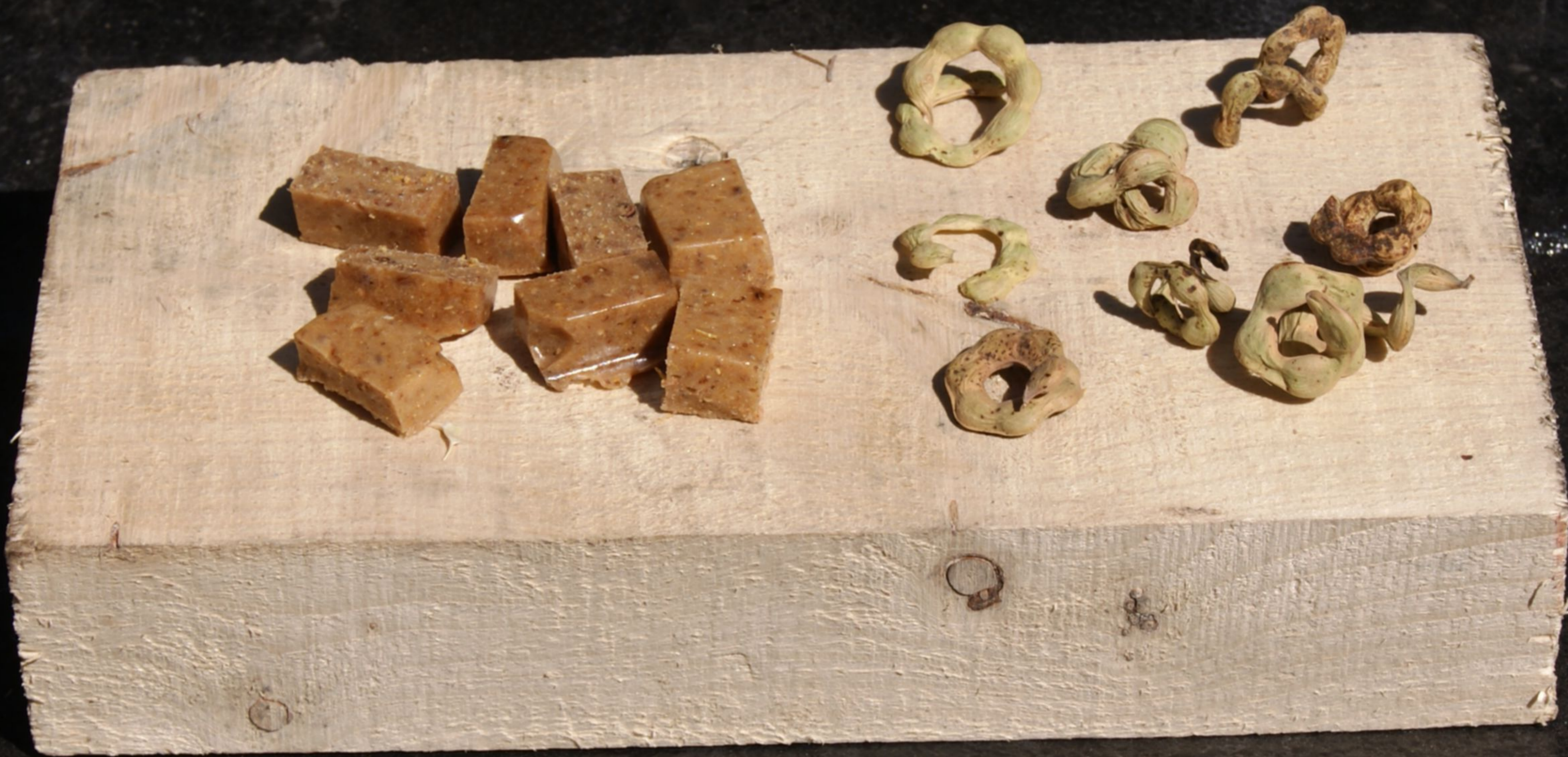

Supplement: Supplementary file 2 — Preparation of 8 pieces of the gelatin-based baits mixed with grinded pods of the camel thorn tree (type 1, left) mixed with 8 control baits (umbrella thorn tree pods, right - control bait 1) to be homogenously mixed in the food tray in bating study 2 (PDF 848 kb) [file 10344_2018_1220_MOESM2_ESM.pdf]

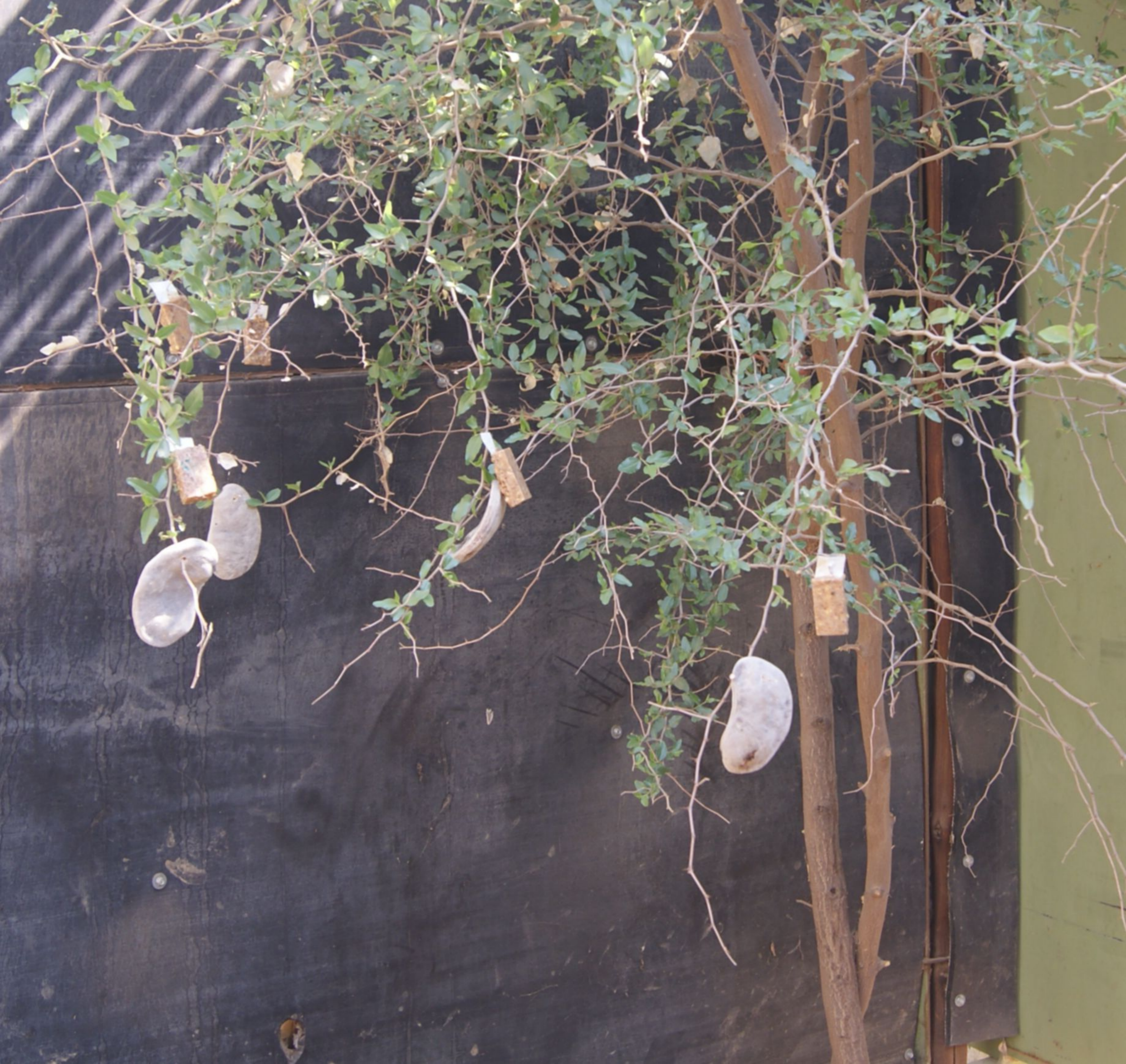

Supplement: Supplementary file 3 — Experimental baits pierced on the thorns of the acacia bushes for bait-uptake by free-roaming Kudu within the enclosure (PDF 865 kb) [file 10344_2018_1220_MOESM3_ESM.pdf]

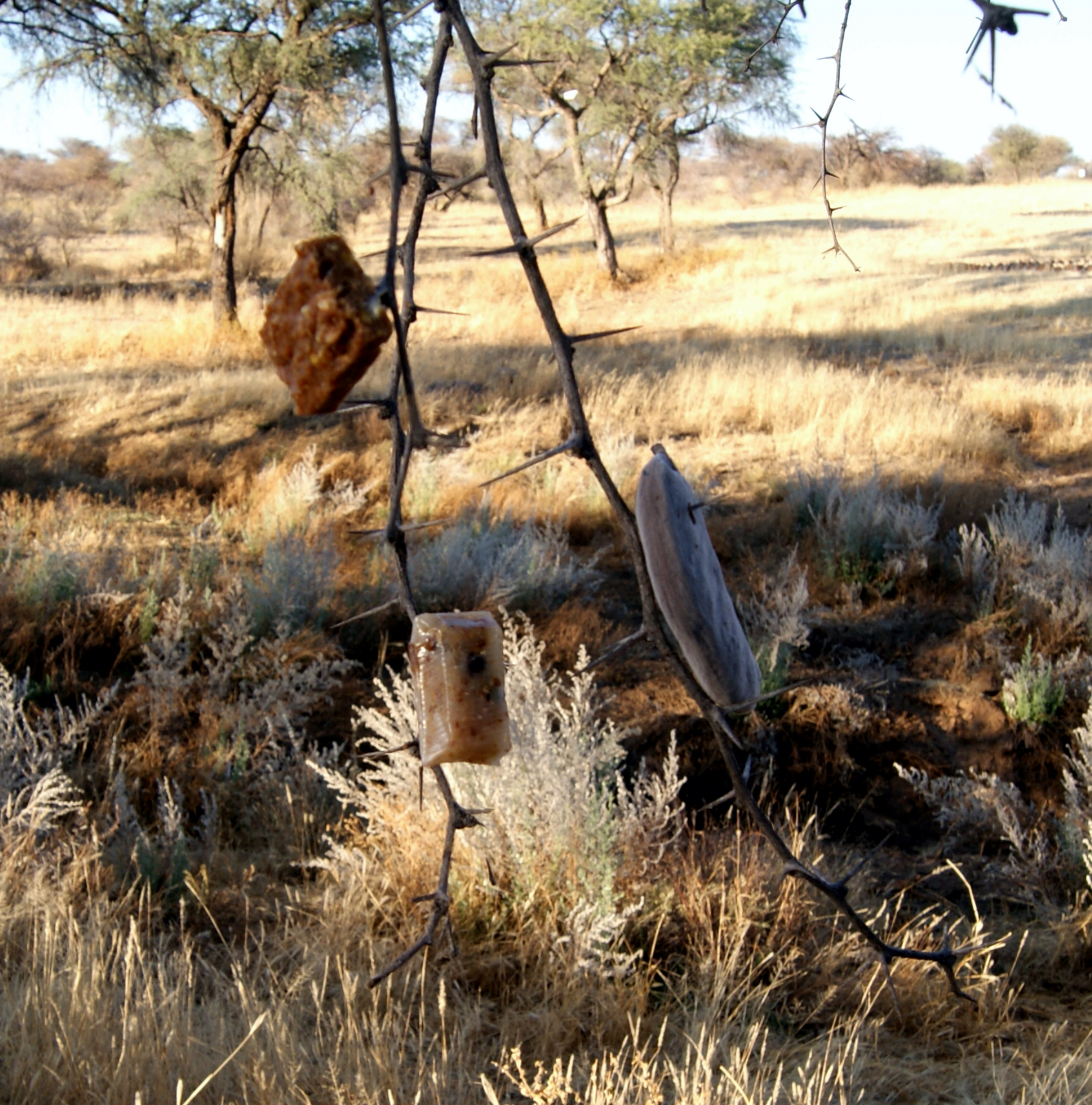

Supplement: Supplementary file 4 — Placebo baits were pierced on the thorns of an acacia tree (left) located at a waterhole close to the experimental facility. (PDF 1448 kb) [file 10344_2018_1220_MOESM4_ESM.pdf]

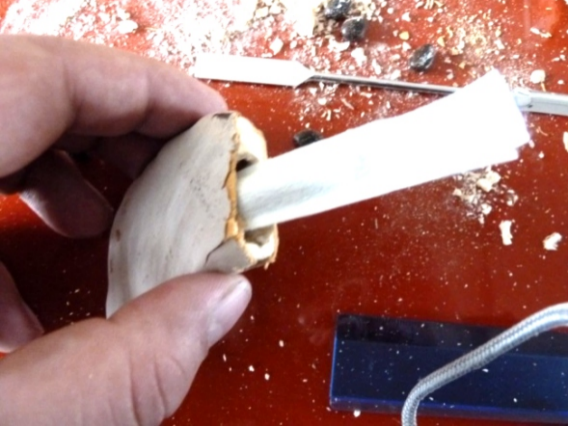

Supplement: Supplementary file 5 — The vaccine sachet from biodegradable foil placed in the camel thorn tree pod (control bait 2) (PDF 133 kb) [file 10344_2018_1220_MOESM5_ESM.pdf]
