# Supplementary material for: Baiting studies on oral vaccination of the greater kudu (Tragelaphus strepsiceros) against rabies
Source: Eur J Wildl Res. 2018 Oct 9;64(6):62. doi: 10.1007/s10344-018-1220-z (PMC7088030; doi:10.1007/s10344-018-1220-z)
Supplement: Supplementary file 6 — (PDF 147 kb) [file 10344_2018_1220_MOESM6_ESM.pdf]

Online Resource 6 Number of baits disappeared after selected periods in pens where sachet loaded baits were offered free-of-choice (h – hours). The baits were pierced on the thorns of acacia shrubs within the outdoor section of the enclosure. Experimental baits used: original camel thorn tree pods (control bait 2), apple-flavoured corn meal (type 3), grinded camel thorn tree pods mixed with gelatine (type 1 – dipped or poured)

**Group**	**Animals (N)**	**Bait type**	**Number of baits present**			
			**0h**	**3h**	**8h**	**24h**
A	2	Control bait 2	10	1*	-	-
B	3	3	15	11	5	1
C	2	1 (dipped)	10	10	10	10
D	3	1 (poured)	15	1	1	1

* - one bait left was found on the ground and removed from the pen
